# Left vocal cord paralysis in a patient with pleuroparenchymal fibroelastosis

**DOI:** 10.1093/omcr/omad108

**Published:** 2023-10-23

**Authors:** Reimi Mizushima, Rena Tamenaga, Taro Kufukihara, Yukihisa Takeda, Yusuke Watanabe, Hiroyuki Nakamura, Kazutetsu Aoshiba

**Affiliations:** Department of Respiratory Medicine, Tokyo Medical University Ibaraki Medical Center, Ibaraki, Japan; Department of Respiratory Medicine, Tokyo Medical University Hospital, Tokyo, Japan; Department of Respiratory Medicine, Tokyo Medical University Ibaraki Medical Center, Ibaraki, Japan; Department of Respiratory Medicine, Tokyo Medical University Hospital, Tokyo, Japan; Department of Respiratory Medicine, Tokyo Medical University Ibaraki Medical Center, Ibaraki, Japan; Department of Respiratory Medicine, Tokyo Medical University Hospital, Tokyo, Japan; Department of Respiratory Medicine, Tokyo Medical University Ibaraki Medical Center, Ibaraki, Japan; Department of Respiratory Medicine, Tokyo Medical University Ibaraki Medical Center, Ibaraki, Japan; Department of Infection Prevention and Control, Tokyo Medical University Hospital, Tokyo, Japan; Department of Respiratory Medicine, Tokyo Medical University Ibaraki Medical Center, Ibaraki, Japan; Department of Respiratory Medicine, Tokyo Medical University Ibaraki Medical Center, Ibaraki, Japan

## CLINICAL IMAGE

**Figure 1:**
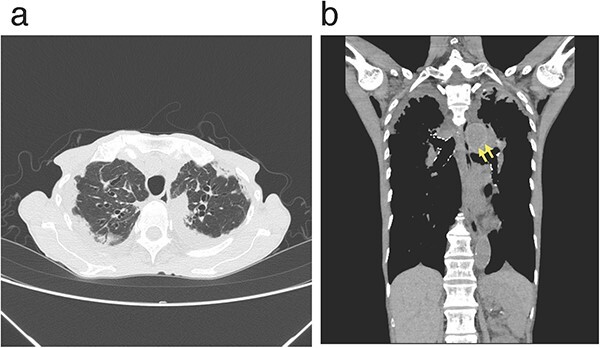
Chest computed tomography images displaying pleural thickening with pulmonary fibrosis, volume loss, and traction bronchiectasis in the bilateral upper lobes (**a**, transverse view of lung window image; **b**, coronal view of mediastinal window image). Note that the aortic arch and left pulmonary artery are in close contact (*arrows*).

A 75-year-old female patient was referred to our hospital by an otolaryngologist with a history of voice hoarseness resulting from left-sided vocal cord paralysis (VCP) and exertional dyspnea. Chest X-ray and chest computed tomography (CT) (Fig. 1) revealed bilateral upper-lobe dominant pleural thickening with subpleural fibrosis, volume loss, and upward tracheobronchial tree retraction; which are radiologic features consistent with pleuroparenchymal fibroelastosis (PPFE) [1]. She also reported symptoms such as breathlessness, weight loss (lost 10 kg over a year), and plathythorax, i.e. anteroposterior chest flattening; which were suggestive of PPFE. A reduction in forced vital capacity (51% predicted) with a restricted pattern was observed in the pulmonary function tests. Blood tests revealed the absence of autoimmune antibodies and nonelevated serum levels of Krebs von den Lungen-6. No other underlying causes of VCP, such as thyroid or lung cancer, mediastinal lymphadenopathy, or aortic aneurysms, were identified. The patient was diagnosed with idiopathic PPFE accompanying left VCP. PPFE is a rare lung disease characterized by predominant upper-lobe fibroelastosis and pleural thickening, with frequent complications of pneumothorax and pneumomediastinum [1]. Six cases of PPFE with VCP, including the present case, with symptoms of hoarseness [2–6] and occasionally aspiration pneumonia [3, 6] have been reported. VCP occurred predominantly on the left side in five out of the six cases, probably due to a longer intrathoracic path of the left recurrent laryngeal nerve (RLN) [2, 3, 5, 6]. Several mechanisms that have been proposed for the underlying VCP in PPFE are: RLN stretching or retraction due to its chest wall adhesion resulting from fibrosis [3], tracheobronchial tree distortion leading to RLN traction or compression [5, 6], and left RLN compression in the aortopulmonary window where the aortic arch and left pulmonary artery are in close contact (Fig. 1b) [5, 6]. PPFE should be considered as a differential diagnosis for VCP.

## Supplementary Material

English_editing_certificate_omad108

## Data Availability

The data supporting the findings of this study are available from the corresponding author upon request; they are not publicly available due to privacy or ethical restrictions.
